# Tailored Practical Simulation Training in Robotic Surgery: A New Educational Technology

**DOI:** 10.1016/j.atssr.2023.05.024

**Published:** 2023-06-22

**Authors:** Takashi Eguchi, Masatoshi Shimura, Shuji Mishima, Daisuke Hara, Shunnichiro Matsuoka, Hirotaka Kumeda, Kentaro Miura, Kazutoshi Hamanaka, Kimihiro Shimizu

**Affiliations:** 1 Division of General Thoracic Surgery, Department of Surgery, Shinshu University School of Medicine, Matsumoto, Japan

## Abstract

**Purpose:**

We aimed to create a tailored robotic surgery training program that addresses current challenges, enhances patient outcomes, and focuses on skills, knowledge, and strategy.

**Description:**

This study assesses the strengths and weaknesses of existing robotic surgery training methods and proposes a personalized simulation training approach for specific surgical situations. The program emphasizes technical and manual skill development, a robust medical knowledge foundation, and strategic planning using the development, demonstration, discussion, and sharing (3DS) concept.

**Evaluation:**

Traditional training challenges, such as on-the-job training, animal models, and cadavers, are identified and addressed. The proposed tailored practical simulation training provides a cost-effective, realistic surgical experience, allowing surgeons to learn in a controlled environment and improving patient outcomes.

**Conclusions:**

This comprehensive training program aims to enhance surgical outcomes and patient care in the rapidly evolving field of robotic surgery. Continuous education, industry collaboration, and knowledge sharing are vital to staying current with advances and optimizing surgical techniques and practices.

## Visual Abstract

**Figure:**
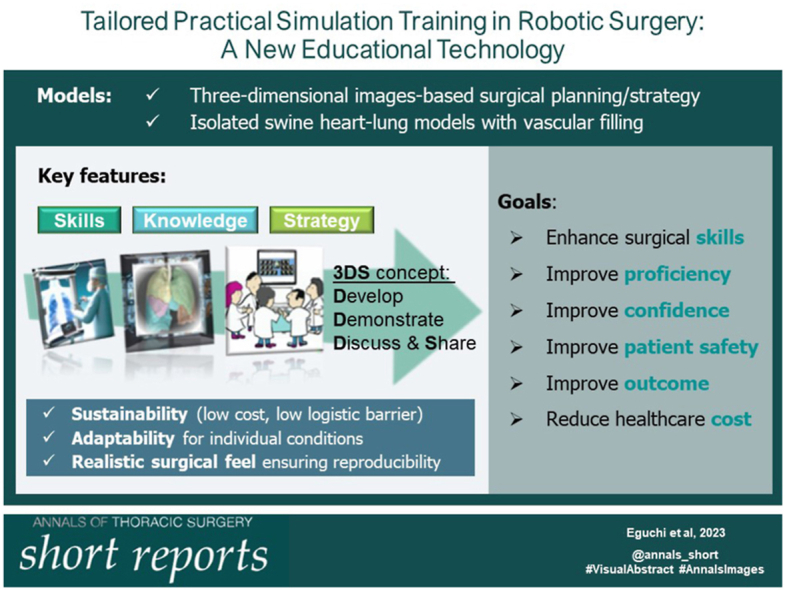

## Technology

**Figure 1:**
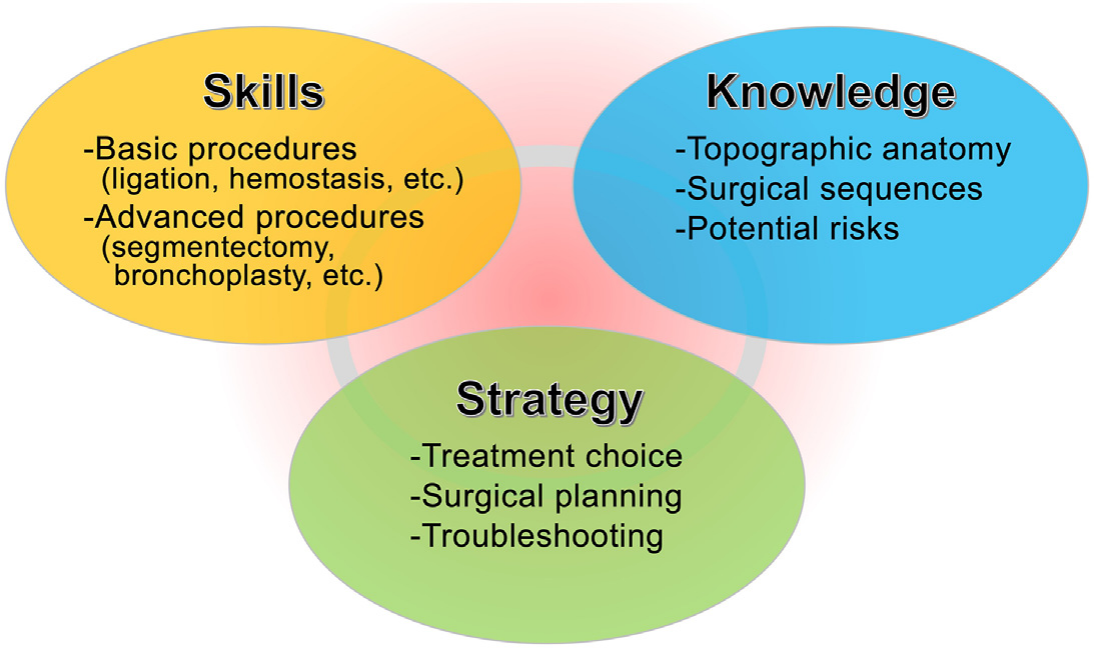
Three elements for surgical treatment success.

Robotic surgery has transformed the surgical field, offering exceptional control, precision, and accuracy for complex procedures. Specialized training is crucial for the safe and effective use of this technology. This study evaluates the strengths and weaknesses of current robotic surgery training programs and proposes a comprehensive, tailored training approach addressing skills, knowledge, and strategy (Figure 1). We aim to improve surgical outcomes and to provide high-quality patient care in this rapidly evolving field. Video 1 offers a visual summary of the concept and article.

## Technique

**Figure 2:**
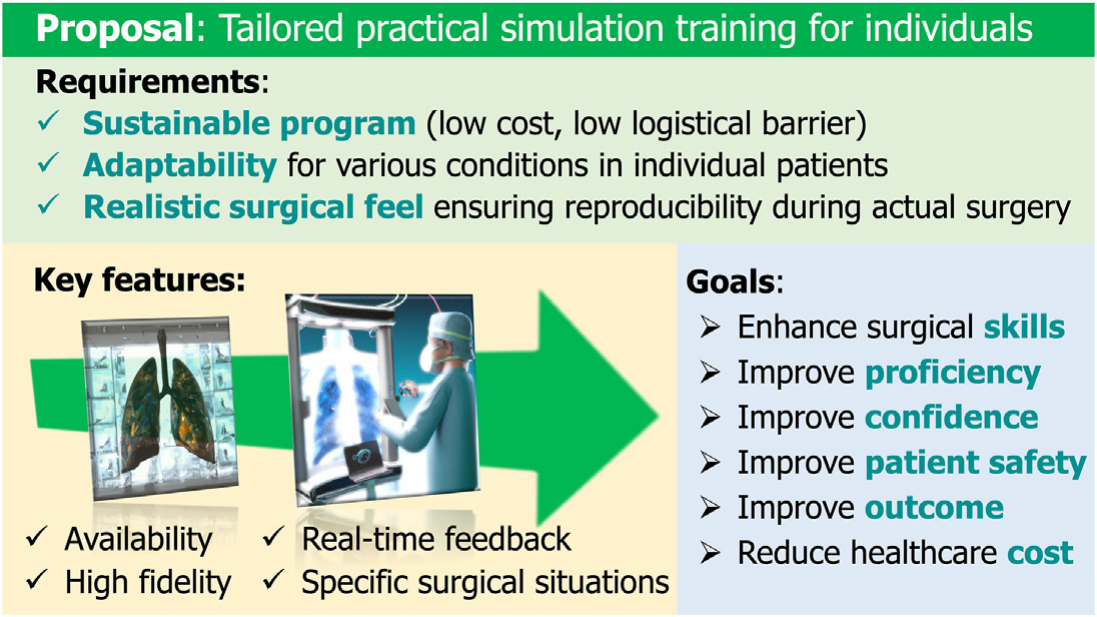
Tailored practical simulation training for specific surgical situations in individual patients.

Tailored practical simulation training presents a personalized approach to robotic surgery education, focusing on individual anatomic features and specific surgical scenarios. Addressing the 3 essential elements of surgical success—skills, knowledge, and strategy—this training method provides sustainable, adaptable, and realistic experiences. Surgeons can develop expertise through personalized, controlled simulations resembling individual patient situations (Figure 2). This section explores the technologic aspects of tailored practical training simulations based on these fundamental concepts.

### Skills

#### Manual and Technical Skills

Robot-assisted thoracic surgery (RATS) demands the development and maintenance of both technical and manual skills, from basic techniques like grasping, hemostasis, and ligation to complex, situation-specific processes needed in challenging scenarios. RATS offers advantages, such as improved visualization and enhanced dissection capabilities, but also has limitations, like the lack of tactile sensation. Surgeons must rely on visual feedback and indirect cues. For successful robotic surgery, effective training is essential for using the robotic platform's features and adapting to various surgical situations. The Table presents an overview of specific techniques, related surgical conditions, recommended training, and associated characteristics, including anatomic consistency, cost, and other factors.

**Table:** Characteristics and Target Maneuvers of Simulation Training Models

Characteristics/Target Maneuvers	Explanation/Corresponding Surgical Situations in Each Maneuver	Simulation Training Models					
		VR	Poultry Tissue^a^	Artificial Organ	Isolated Swine Heart-Lung With Vascular Filling	Anesthetized Live Swine	Cadaver^b^
Characteristics		Scale of evaluation: −, negative; +, intermediate or acceptable; ++ , positive					
Anatomic consistency	Poor (negative) or good (positive)	−	−	−	+	+	++
Surgical feeling consistency	Poor (negative) or good (positive)	−	+	+	++	++	+
Cost performance (eg, initial purchase, running cost)	Expensive (negative) or inexpensive (positive)	−	++	++	+	−	−
Preparation/support (time consumption, required staff)	Inefficient (negative) or efficient (positive)	++	++	++	+	−	−
Technical complexity (barrier to beginners)	Complicated (negative) or simple (positive)	++	++	++	++	−	+
Target maneuvers		Scale of evaluation: −, not applicable or unsuitable; +, acceptable; ++, suitable					
Basic, simple maneuvers							
Grasping/gripping tissue	Basic dissection	+	++	++	++	++	++
Dividing tissue with cauterization	Basic dissection	+	++	+	++	++	++
Hemostasis	Basic dissection	+	−	−	+	++	−
Ligation	Dividing vessels	+	+	+	++	++	++
Suturing	Suturing visceral pleura	+	+	+	++	++	++
Stapling	Dividing lung parenchyma and vessels	−	−	+	++	++	++
Retraction	Retraction of lung	+	++	++	++	++	++
Advanced, combined maneuvers							
Cross-countertraction	Lung retraction for dividing the central intersegmental plane	−	+	−	++	++	++
Scraping	Dissection of bronchial surrounding tissue	−	−	−	++	++	++
Sliding and dividing	Dividing central intersegmental plane	−	+	−	++	++	++
Soft grasping/hard gripping	Hilar and segmental lymph node dissection	−	++	+	++	++	++
Distal stump lifting	Dissection of tissue behind the distal vascular and bronchial stumps	−	−	−	++	++	++
Advanced suturing	Bronchoplasty	−	−	−	++	++	++

VR, virtual reality.

a Poultry that is available in grocery stores (eg, wing-tip, liver).

b Cadaver training without vascular filling.

#### Challenges in Surgical Training Methods

Traditional surgical training follows the "see one, do one, teach one" concept, but this is less effective for minimally invasive surgeries like RATS because trained physicians cannot fully control the movements of inexperienced physicians through small ports. The proposed "see one, simulate many, do one competently, teach everyone" concept emphasizes simulation-based training and practice, allowing surgeons to develop skills in a controlled environment before performing patient procedures.^1^ Animal models and cadavers pose challenges; cadavers provide consistent anatomy but lack realistic surgical sensation because of the absence of vascular filling. Some cadaver models include vascular filling, but they are expensive and time-consuming.^2^

#### Reality and Adaptability

Realistic surgical experiences are vital for skill development, ensuring that surgeons successfully apply their learning to actual procedures. A training model that closely mimics real surgical experiences provides a more accurate representation of tissues and structures encountered. Adaptability to various conditions is crucial for optimal patient outcomes. Training methods should be tailored, with practical simulations allowing surgeons to practice different techniques and to adapt to multiple situations during surgery.

### Knowledge

#### Topographic Anatomy

Learning topographic anatomy is critical in robotic surgery training. Although robotic surgery provides a magnified and enlarged local view of the surgical site, understanding the anatomy from the broad scope of the entire chest during RATS can be challenging for surgeons. Three-dimensional computed tomography (3D-CT) is essential for understanding the 3D relationships between the surgical site and surrounding structures.^3^, ^4^, ^5^ This understanding is critical for safe and effective surgical dissection. However, 3D-CT has potential drawbacks, such as time-consuming image preparation and difficulty in obtaining realistic simulation images.

#### Sequence of Surgical Procedures

The sequence of surgical procedures is another important aspect of robotic surgical knowledge.^6^ Robotic surgery–specific procedures require specific knowledge and skills, such as rolling-in, targeting, rolling-out, robotic stapling, and robotic suturing.

#### Potential Risks and Complications

Surgeons should be aware of the risks and complications associated with robotic surgery. Although robotic surgery has a low risk of catastrophic events, potential risks must be carefully considered and addressed.^7^^,^^8^ Surgeons must make rapid and appropriate decisions in the event of unexpected complications.

### Strategy

#### Surgical Planning With the Development, Demonstration, Discussion, and Sharing (3DS) Concept

**Figure 3:**
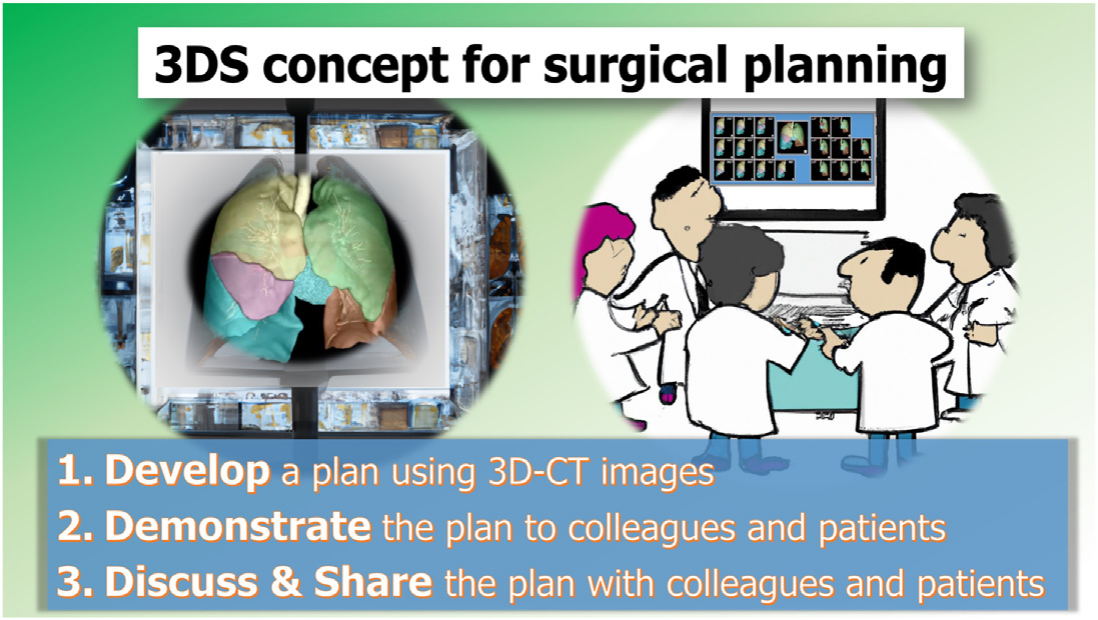
The development, demonstration, discussion, and sharing (3DS) concept for surgical planning. (3D-CT, 3-dimensional computed tomography.)

Surgical planning is a key aspect of effective surgical strategy. The 3DS concept serves as a useful framework for surgical planning (Figure 3). Using 3D-CT images, surgeons can develop a 3D model of the surgical site, allowing them to better understand the relationships between structures and to plan the surgical approach.^4^^,^^6^ Surgeons can then demonstrate the plan to the surgical team and discuss it in detail to ensure that everyone is in agreement. Finally, the plan should be shared with colleagues and patients to ensure that everyone is fully informed and prepared.

#### Personalized Medicine

A successful surgical strategy tailors the treatment approach to each patient's unique needs. The tailored practical training simulation integrates personalized medicine by considering factors like patient age, medical history, and overall health in planning a surgical approach.^9^

#### Troubleshooting

The ability to address unexpected situations during surgery is vital for ensuring the best possible patient outcomes. Surgeons must be prepared to make rapid, appropriate decisions in these situations. To ensure preparedness, the surgical team should consider conducting a didactic course for beginners and role-play scenario–based simulation training for intraoperative catastrophes, such as pulmonary artery injury.

## Clinical Experience

To address the limitations of traditional on-the-job training and to ensure sustainable training programs, we propose a tailored practical simulation training approach for specific surgical situations in individual patients at Shinshu University Hospital. This method emphasizes the importance of realistic surgical feel and personalized simulation tailored to each patient's unique needs. By offering individual simulations for distinct surgeries, surgeons can gain experience in a personalized and controlled environment, enhance their skills, and improve patient outcomes.

We used materials such as chicken meat, artificial organs, and isolated swine heart-lung models with vascular filling (Hybrid Lung; Johnson & Johnson K.K.) for training. Chicken meat offers a lower level of surgical realism but is more affordable and superior to virtual reality training. The Hybrid Lung model, although more expensive, provides a realistic surgical feel akin to living animals and cadavers. These models can be applied in various surgical procedures, balancing sustainability, adaptability, and realism.

The Hybrid Lung is considered the core training method in our tailored practical simulation training system, providing a highly realistic surgical experience, adaptability to various surgical situations, low logistical barriers, and relatively high availability with industry partner support. Vascular filling maintains a realistic surgical feel for hilar dissection in this model. Video 2 demonstrates a detailed maneuver to dissect the left anterior segmental branch of the pulmonary artery, showcasing how the training reproduces actual surgery. The absence of background movement due to heartbeat and respiration enables surgeons to concentrate on procedures and to accelerate learning.

At our institution, 3D-CT plays a crucial role in surgical planning. We adopted a novel 3D-CT workstation (Revoras; Ziosoft, Inc) that offers semiautomated measurements and surgeon-oriented 3D views for understanding surgical sequences, resected bronchovascular stumps, and intersegmental plane shapes.^4^ Combining 3D-CT and simulation training allows reproduction of specific surgical fields, including visualization, port setting, and hilar dissection direction, before surgery. This integration of 3D-CT–based planning with surgical practice enhances surgical precision and patient outcomes.

## Comment

The tailored practical simulation and training approach holds promise for enhancing robotic surgery education. This approach aims to improve patient care by overcoming traditional surgical training limitations by addressing essential elements of surgical success (skills, knowledge, and strategy) and emphasizing personalized, sustainable, adaptable, and realistic training methods.

Important aspects to consider in implementing tailored practical simulation training include the following:

• Ongoing technologic advancements: Training methods must be updated as surgical and simulation technologies evolve. Virtual reality, augmented reality, artificial intelligence, and machine learning can enhance training realism, personalization, and effectiveness.
• Collaboration and knowledge sharing: Success relies on effective collaboration between institutions, industry partners, and surgical teams. Developing standardized training protocols and curricula can promote consistency and widespread adoption in surgical training.
• Validation and assessment: Rigorous validation studies should evaluate the impact of this approach on surgical performance, patient outcomes, and overall surgical quality.
• Cost-effectiveness and resource allocation: Financial implications of implementing tailored practical simulation training must be considered. Although initial investments are required, the long-term benefits of improved surgical outcomes may offset costs.

A study evaluating surgical residents' and fellows' training progress using a virtual reality–based simulation found that junior residents achieved compression more efficiently than senior trainees.^10^ This highlights the importance of adapting training methods to trainees’ experience levels and skills. Incorporating virtual reality and other simulation technologies can enhance surgical education effectiveness and efficiency. Ongoing research and evaluation of these training methods are crucial for continued success and improvement.

In conclusion, tailored practical simulation training represents a promising approach to robotic surgery education, potentially revolutionizing surgical training and improving patient care. Ongoing refinement and validation will be essential as technology advances and the field evolves.

Moving forward, our next step is to conduct a comprehensive study to further investigate and validate the effectiveness of this tailored practical simulation training method. This study will evaluate performance metrics, such as surgical procedure times, complication rates, and patient outcomes, before and after training. By quantitatively assessing these aspects, we aim to provide concrete evidence supporting our training method's benefits. We believe this forthcoming study will contribute significantly to the existing body of knowledge in robotic surgery training, bringing us closer to our ultimate goal of improving surgical outcomes and enhancing patient care.

## Freedom of Investigation

The technology used in this study was purchased. The authors had full control of the study design, methods, outcome parameters, data analysis, and production of the written report.

## Disclaimer

The Society of Thoracic Surgeons, The Southern Thoracic Surgical Association, and *The Annals of Thoracic Surgery* neither endorse nor discourage the use of the new technology described in this article.
